# fMRI-Based Brain Responses to Quinine and Sucrose Gustatory Stimulation for Nutrition Research in the Minipig Model: A Proof-of-Concept Study

**DOI:** 10.3389/fnbeh.2018.00151

**Published:** 2018-07-24

**Authors:** Nicolas Coquery, Paul Meurice, Régis Janvier, Eric Bobillier, Stéphane Quellec, Minghai Fu, Eugeni Roura, Hervé Saint-Jalmes, David Val-Laillet

**Affiliations:** ^1^INRA, INSERM, Univ Rennes, Nutrition Metabolisms and Cancer, NuMeCan, Rennes, France; ^2^IRSTEA, UR OPAALE, Rennes, France; ^3^Centre for Nutrition and Food Sciences, Queensland Alliance for Agriculture and Food Innovation, The University of Queensland, St. Lucia, QLD, Australia; ^4^CLCC Eugène Marquis, Inserm, LTSI-UMR 1099, Université de Rennes, Rennes, France

**Keywords:** fMRI, brain, pig, nutrition, gustatory stimulation, visual stimulation, hedonism

## Abstract

The minipig model is of high interest for brain research in nutrition and associated pathologies considering the similarities to human nutritional physiology, brain structures, and functions. In the context of a gustatory stimulation paradigm, fMRI can provide crucial information about the sensory, cognitive, and hedonic integration of exteroceptive stimuli in healthy and pathological nutritional conditions. Our aims were (i) to validate the experimental setup, i.e., fMRI acquisition and SPM-based statistical analysis, with a visual stimulation; (ii) to implement the fMRI procedure in order to map the brain responses to different gustatory stimulations, i.e., sucrose (5%) and quinine (10 mM), and (ii) to investigate the differential effects of potentially aversive (quinine) and appetitive/pleasant (sucrose) oral stimulation on brain responses, especially in the limbic and reward circuits. Six Yucatan minipigs were imaged on an Avanto 1.5-T MRI under isoflurane anesthesia and mechanical ventilation. BOLD signal was recorded during visual or gustatory (artificial saliva, sucrose, or quinine) stimulation with a block paradigm. With the visual stimulation, brain responses were detected in the visual cortex, thus validating our experimental and statistical setup. Quinine and sucrose stimulation promoted different cerebral activation patterns that were concordant, to some extent, to results from human studies. The insular cortex (i.e., gustatory cortex) was activated with both sucrose and quinine, but other regions were specifically activated by one or the other stimulation. Gustatory stimulation combined with fMRI analysis in large animals such as minipigs is a promising approach to investigate the integration of gustatory stimulation in healthy or pathological conditions such as obesity, eating disorders, or dysgeusia. To date, this is the first intent to describe gustatory stimulation in minipigs using fMRI.

## Introduction

The pig and minipig models are now recognized as one of the most prominent large animal model for human nutritional physiology (Roura et al., 2016; Roura and Fu, 2017) and neuroimaging studies (Sauleau et al., 2009; Clouard et al., 2012b; Val-Laillet et al., 2015). Given the exponential use of magnetic resonance imaging in human, many efforts have to be made in order to adapt MRI setup to the pig and minipig specificities. Regarding brain investigation with functional MRI (fMRI) and, to our knowledge, only few studies were performed in pigs and minipigs, including different kinds of stimulation paradigms and stimuli, such as visual (Fang et al., 2006; Gizewski et al., 2007), somatosensory (Fang et al., 2005; Duhaime et al., 2006), pharmacological (Mäkiranta et al., 2002), and deep-brain stimulations (Min et al., 2012; Knight et al., 2013; Paek et al., 2015; Gibson et al., 2016; Settell et al., 2017). To date, no study has been performed with fMRI to explore the brain responses to gustatory stimulations for nutrition research.

Olfactory and gustatory stimulations have already been widely investigated in the pig and minipig models. Roura and Fu (2017) and Val-Laillet (2018) have recently provided complete reviews on this topic and the scope of the methodologies used for this purpose come from ethology (Clouard et al., 2012a,c; Clouard and Val-Laillet, 2014) to electrophysiology (Danilova et al., 1999) and nuclear imaging, such as single photon emission computed tomography (SPECT) or positron emission tomography (PET; Clouard et al., 2012a, 2014b; Val-Laillet et al., 2015; Val-Laillet, 2018). These approaches provide either information about a long-term integration of the stimulation, i.e., behavior or nuclear imaging, or a direct but local impact, i.e., electrophysiology. Furthermore, nuclear imaging, usually performed with an acquisition time frame of several minutes, can only inspect the global brain changes, such as in brain perfusion for SPECT with hexa-methyl-propyl-amineoxime (HMPAO) detection, or glucose metabolism for PET with fluoro-deoxyglucose detection. fMRI solely can provide information on the acute response (15–20 s) to a repeated single and short stimulation in a global brain analysis, making this methodology of high interest for nutritional studies. Moreover, different kinds of stimulations and their controls can be investigated during the same imaging session, contrary to paradigms using nuclear imaging, which significantly increases the methodological strength and statistical comparison power between stimulations, in addition to temporal resolution, and decreases the costs and constraints for animal experimentation on large animals.

The couple pleasant vs. aversive stimulation is currently gaining more attention for nutritional studies in human. Indeed, this approach is being used to decipher the organization of the gustatory cortex, e.g., the insular cortex (Rudenga et al., 2010; Dalenberg et al., 2015) and the associated brain areas (Zald et al., 2002; van den Bosch et al., 2014; Field et al., 2015). This approach is also used to investigate the impact of aging (Hoogeveen et al., 2015) or pathologies, such as obesity (Szalay et al., 2012), on those brain areas. Besides species differences in terms of taste detection and integration between human and the pig or minipig model (Roura and Fu, 2017), the pleasant vs. aversive impact of compounds have been already investigated with behavioral exploration and nuclear imaging in pigs (Clouard et al., 2012b,c) but not with fMRI.

In this study, we aim to validate in the minipig model a fMRI-based analysis of brain responses to sucrose vs. quinine stimulation as a model of pleasant vs. aversive stimulation paradigm. For this purpose, we first validate the experimental and analysis setup with a visual stimulation based on a previous study (Gizewski et al., 2007). This first attempt of fMRI analysis of gustatory stimulation should be of high interest for nutritional studies for basic research and pre-clinical purposes.

## Materials and Methods

### Animals

Experiments were conducted in accordance with the current ethical standards of the European Community (Directive 2010/63/EU), Agreement No. C35-275-32, and Authorization No. 35-88. The Regional Ethics Committee in Animal Experiment of Brittany has validated and approved the entire procedure described in this paper (Project No. 2015051312053879). A total of six 1-year-old 30-kg male Yucatan minipigs were used in this study. The pigs were housed in individual pens (150 cm × 60 cm × 80 cm) and had free access to water. A chain was suspended in each pen to enrich the environment of the animals and fulfill their natural disposition to play. The room was maintained at ∼24°C with a 13:11-h light–dark cycle.

### Anesthesia

Pre-anesthesia was performed with an intramuscular injection of ketamine (5 mg/kg – Imalgene 1000, Merial, Lyon, France) in overnight-fasted animals. Isoflurane inhalation (Aerane 100 ml, Baxter SAS, France) was used to suppress the pharyngotracheal reflex and then establish a surgical level of anesthesia, 3–5 and 2–3% v/v, respectively. After intubation, anesthesia was maintained with 2.5% v/v isoflurane and mechanical respiration allowed adjustment of respiratory frequency at 20 breathing/minute with a tidal volume of 450 ml. Cotton wool with an additional headset were used to conceal the animal’s ears.

### Visual and Gustatory Stimulation

For visual and gustatory stimulation, we used a custom-made stimulation apparatus, which was located outside the magnet-shielded room (5-m distance) and used to deliver both visual and gustatory stimulations upon synchronization with the MRI system.

#### Visual Stimulation

Both eyes were entirely covered by an opaque cap. The stimulation apparatus produced the visual stimulation that was conducted to the animal’s right eye cap with an optical fiber. Visual simulation consisted of flashlight at a frequency of 8 Hz. A block paradigm was used: 20-s stimulation ON, 20-s stimulation OFF, repeated 15 times. The entire stimulation protocol duration was about 10 min.

#### Gustatory Stimulation

Animals were equipped with an oral apparatus allowing gustatory stimulation as previously described (Clouard et al., 2012a) and consisting of three tubes, one for each solution, with an additional circular tube for continuous aspiration of liquid. Quinine (10 mM, Q1125-25G, Sigma-Aldrich, St. Quentin Fallavier, France) and sucrose (5%, S/8560/65, Fisher Chemical, Leics, United Kingdom) were solubilized in artificial saliva (Hellekant et al., 1997). In order to obtain the highest brain responses for each stimulation, three blocks of stimulation were performed in the following order, from the least to the most persistent solution in mouth: control stimulation with artificial saliva, sucrose stimulation, and quinine stimulation, each stimulation being repeated 15 times. Each stimulation consisted of oral stimulation (5 s, 24 mL/min), 25-s pause, rinse with artificial saliva (15 s, 24 mL/min), and pause (15 s). The entire stimulation protocol duration was about 45 min.

### MRI Image Acquisition

Image acquisition was performed on a 1.5-T magnet (Siemens Avanto) at the Rennes Platform for Multimodal Imaging and Spectroscopy. Acquisitions were performed using a combination of coils (Body and Spine matrix coils) for optimized signal to noise ratio acquisition. *T1-weighted anatomical image acquisition*: a MP-RAGE sequence was adapted for adult minipig anatomy (1.2 mm × 1.2 mm × 1.2 mm, NA = 2, TR = 2400 ms, TE = 3.62 ms, TI = 854 ms, FA = 8°, acquisition duration 15 min). *BOLD signal acquisition*: an echo planar imaging sequence was adapted for minipig head geometry (TR/TE: 2500/40 ms, FA: 90°, voxel size: 2.5 mm × 2.5 mm × 2.5 mm). The first four acquired volumes were excluded for the data analysis, meaning that no stimulation was performed during this period.

### Data Analysis and Statistical Image Analysis

Data analysis was performed with SPM12 (version 6906, Wellcome Department of Cognitive Neurology, London, United Kingdom). After slice timing correction, realignment, and spatial normalization on a pig brain atlas (Saikali et al., 2010), images were smoothed with a Gaussian kernel of 4 mm. *Voxel-based statistic*: first-level (within-individual contrast) and second-level (within-group contrast) statistics were assessed with a threshold set at *p* < 0.05 to produce the brain maps of activation. No suprathreshold voxels were detected with FDR correction at *p* < 0.05. *ROI-based statistic*: anatomical ROIs from the Saikali pig atlas (Saikali et al., 2010) were extracted and ROI-based statistic was performed using the Marsbar toolbox with a uncorrected *p*-value threshold set at 0.05 for the whole ROI. Statistical analysis was performed either on single stimulation, e.g., changes compared to baseline, or between stimulations differences. Data from only five minipigs were used for visual analysis due to a complication during acquisition of the visual stimulation in one animal.

## Results

### fMRI Setup and Statistical Analysis Validation With Visual Stimulation

**FIGURE 1:**
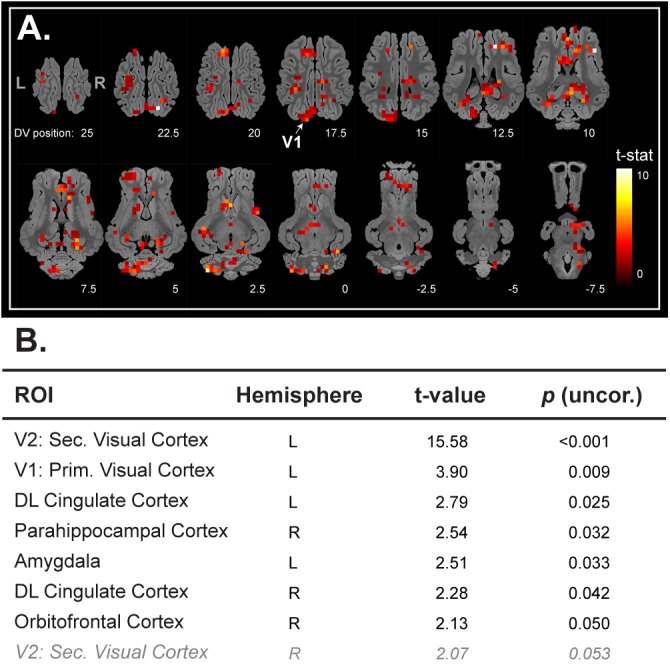
**(A)** Horizontal maps of global brain BOLD responses to a visual stimulation (white flash, 8 Hz). *p*-Value threshold = 0.05; DV, dorsal-ventral position in mm related to the posterior commissure. **(B)** Related ROI-based statistical analysis with ROIs from Saikali atlas (Saikali et al., 2010). ROI abbreviations are detailed at the bottom of the panel **(A)**. Cerebellar brain ROIs were excluded, *p*-values <0.05 are presented in black normal font style, and *p*-value between 0.05 and 0.1 are presented in gray italic.

We first aimed to validate the experimental setup, the acquisition, and the statistical analysis pipeline with a visual stimulation paradigm such as previously described (Fang et al., 2006; Gizewski et al., 2007). We could easily distinguish a significant BOLD response in the left occipital lobe (**Figure 1A**). This cluster was mostly found in the primary visual system V1. The ROI-based statistical analysis confirmed a significant BOLD response in V1 but also in V2 (**Figure 1B**).

### Brain Responses to Artificial Saliva Stimulation

**FIGURE 2:**
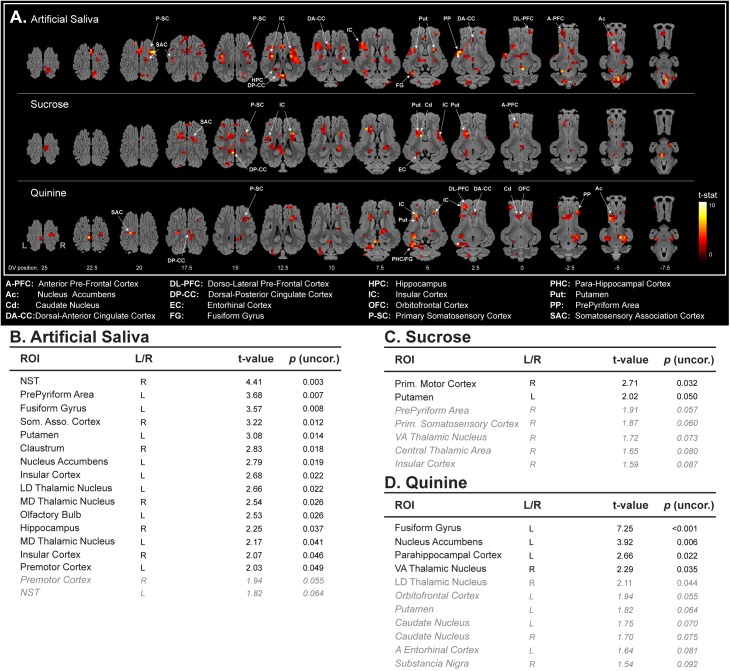
**(A)** Horizontal maps of global brain BOLD responses to artificial saliva, sucrose, quinine, and between sucrose and quinine stimulations. *p*-Value threshold = 0.05; DV, dorsal-ventral position in mm related to the posterior commissure. ROI-based statistical analysis with ROIs from Saikali atlas (Saikali et al., 2010) for **(B)** artificial saliva, and **(C)** sucrose, **(D)** quinine. ROI abbreviations are detailed at the bottom of the panel **(A)**. Cerebellar brain ROIs were excluded, *p*-values <0.05 are presented in black normal font style, and *p*-value between 0.05 and 0.1 are presented in gray italic. Activation and decreased activation are separated by a line and are organize in *p*-value decreasing order.

With artificial saliva stimulation, we could detect a large activation pattern in olfacto-gustatory centers (Rolls, 2005) such as the insular cortex, the prepyriform area, and thalamic regions, but also in other brain areas (**Figure 2A**). The ROI-based statistical analysis validated the increased BOLD responses within these brain regions (**Figure 2B**).

**FIGURE 3:**
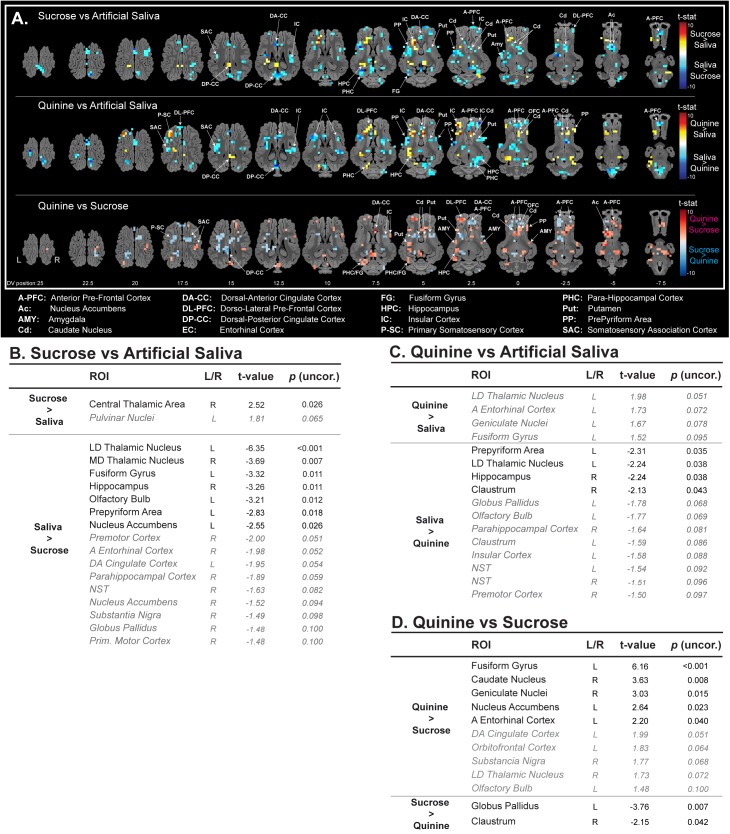
**(A)** Horizontal maps of global brain responses between quinine and artificial saliva, as well as sucrose and artificial saliva stimulations. *p*-Value threshold = 0.05; DV, dorsal-ventral position in mm related to the posterior commissure. ROI-based statistical analysis with ROIs from Saikali atlas (Saikali et al., 2010) for **(B)** sucrose vs. artificial saliva, **(C)** quinine vs. artificial saliva, and **(D)** quinine vs. sucrose (uncorrected *p*-values). ROI abbreviations are detailed at the bottom of the panel **(A)**. Cerebellar brain ROIs were excluded, *p*-values <0.05 are presented in black normal font style, and *p*-value between 0.05 and 0.1 are presented in gray italic.

All stimulations (saliva, sucrose, and quinine) were able to activate, but in different subparts, the primary somatosensory cortex, the somatosensory association cortex, the dorsal posterior cingulate cortex, the insular cortex, and the putamen (**Figure 2A**). For instance, sucrose and quinine stimulation promote brain activation in the most anterior part of the insular cortex, which is in accordance with taste encoding (Rolls, 2016). However, sucrose and quinine stimulations promoted a reduced number of activated voxels in the brain (sucrose: *n* = 174 activated voxels and quinine: *n* = 222 activated voxels) compared with artificial saliva stimulation (*n* = 399 activated voxels), as illustrated in the insular cortex (**Figure 2A**). This is illustrated in the sucrose vs. artificial saliva and in the quinine vs. artificial saliva brain maps in which we detected fewer activated voxels with quinine stimulation than with artificial saliva stimulation (**Figure 3A**). The ROI-based statistical analysis also showed more statistically activated brain regions with artificial saliva stimulation (*n* = 15 for *p* < 0.05, **Figure 2B**) than with sucrose (*n* = 2 for *p* < 0.05) or quinine stimulation (*n* = 4 for *p* < 0.05, **Figures 2C,D**).

### Brain Responses to Sucrose Stimulation

Compared with artificial saliva stimulation, the sucrose stimulation also promoted activations, but in different subparts, in the anterior prefrontal cortex, the dorsal posterior cingulate cortex, a larger activation in the putamen, and an additional activation in the entorhinal cortex and the caudate nucleus (**Figure 2A**).

The ROI-based statistical analysis validated in part the global brain changes and showed an activation of the primary motor cortex and the putamen, a tendency toward activation of five other brain structures (**Figure 2C**).

The sucrose vs. artificial saliva brain map corroborated these observations (**Figure 3A**). Compared to artificial saliva, sucrose promoted a decreased activation in the prepyriform area, the dorsal anterior cingulate cortex, the anterior and the ventral part of the anterior prefrontal cortex, the anterior part of the dorsal lateral prefrontal cortex, the fusiform gyrus, the ventral part of the caudate nucleus, the nucleus accumbens, the amygdala, and both higher activation and decreased activation in the insular cortex and the putamen. The ROI-based statistical analysis of the sucrose vs. artificial saliva stimulation showed a statistical activation only in the central thalamic area and a statistical decreased activation in two thalamic areas, the fusiform gyrus, the hippocampus, the olfactory bulb, the prepyriform area, the nucleus accumbens, and a tendency toward lower activation in nine others brain structures such as, for instance, the entorhinal cortex, the dorsal anterior cingulate cortex, the para-hippocampal cortex, the nucleus accumbens, the substantia nigra, and the globus pallidus (**Figure 3B**).

### Brain Responses to Quinine Stimulation

Compared with artificial saliva stimulation, the quinine stimulation promoted also activations, but in different subparts, in the prepyriform area, the dorsal posterior cingulate cortex, the dorsal lateral prefrontal cortex, a larger activation in the fusiform gyrus, the caudate nucleus, the nucleus accumbens, and an additional activation in the para-hippocampal cortex and the orbitofrontal cortex (**Figure 2A**).

The ROI-based statistical analysis supports in part the global brain changes and showed an activation of the fusiform gyrus, the nucleus accumbens, the para-hippocampal cortex, the ventral anterior thalamic nucleus, a tendency toward activation of seven other brain structures, i.e., the orbitofrontal cortex, the putamen, and the caudate nucleus (**Figure 2D**).

The quinine vs. artificial saliva brain maps corroborated these observations (**Figure 3A**). Compared to artificial saliva, quinine promoted a larger activation in the caudate nucleus, a decreased activation in the ventral part of the anterior prefrontal cortex, the putamen, and both activation and decreased activation in the insular cortex, the prepyriform area, the dorsal lateral prefrontal cortex, the dorsal posterior cingulate cortex, and the hippocampus. The ROI-based statistical analysis of the quinine vs. artificial saliva stimulation only showed a tendency toward activation in four brain structures, i.e., the lateral dorsal thalamic nucleus, the anterior entorhinal cortex, the geniculate nuclei, and the fusiform gyrus; a statistical decreased activation in the prepyriform area, the lateral dorsal thalamic nucleus, the hippocampus, the claustrum, and a tendency toward decreased activation in eight others brain structures, i.e., the globus pallidus, the olfactory bulb, the para-hippocampal cortex, the insular cortex, and the nucleus of the solitary tract (**Figure 3C**).

### Comparison of Brain Responses to Sucrose vs. Quinine Stimulations

The quinine vs. sucrose brain map allowed discriminating the impact of each stimulation. Quinine promoted a higher activation in the dorsal lateral prefrontal cortex, the ventral part of the anterior prefrontal cortex, the prepyriform area, the para-hippocampal cortex, the fusiform gyrus, the hippocampus, the caudate nucleus, the posterior region of the putamen, the nucleus accumbens, and in the amygdala (**Figure 3A**, in red). Sucrose promoted a higher activation in the primary somatosensory cortex, the dorsal anterior cingulate cortex, the dorsal part of anterior prefrontal cortex, and in the putamen (**Figure 3A**, in blue). Note that each stimulation showed higher activation in different subparts of the insular cortex, the anterior prefrontal cortex (larger activation for sucrose stimulation), the putamen (larger activation for sucrose stimulation), and the caudate nucleus (larger activation for quinine stimulation).

The ROI-based statistical analysis of the quinine vs. sucrose stimulation validated in part the global brain changes and showed a higher activation from quinine in the fusiform gyrus, the caudate nucleus, the geniculate nuclei, the nucleus accumbens, and the anterior entorhinal cortex, a tendency toward higher activation of five other brain structures including the dorsal anterior cingulate cortex, the orbitofrontal cortex, the substantia nigra, the lateral dorsal thalamic nucleus, and the olfactory bulb, whereas we could only detect a higher statistical activation with sucrose stimulation in the globus pallidus and the claustrum (**Figure 3D**).

## Discussion

For the first time, this proof-of-concept study describes in the minipig model the brain responses to contrasted gustatory stimulations with fMRI. After validation of our fMRI paradigm with visual stimulation, we were able to detect the brain responses elicited by artificial saliva, sucrose, and quinine oral gustatory stimulations. The statistical analysis also allowed investigating the specificity of quinine stimulation, as an aversive stimulation paradigm, vs. sucrose stimulation, as a pleasant stimulation paradigm.

As a prerequisite, we aimed to validate our experimental setup, image processing, and statistical analysis approaches with a visual stimulation paradigm, as investigated in a previous study (Gizewski et al., 2007). We were able to detect statistically significant brain responses in the contralateral visual cortex but also a trend toward activation in the ipsilateral visual cortex, which is in accordance with previous studies related to visual stimulation in pigs (Fang et al., 2006) and in minipigs (Gizewski et al., 2007). In this proof-of-concept study, the brain responses of five animals were sufficient to detect the brain activation elicited by visual stimulation (uncorrected statistic at a *p*-value level of 0.05). Note that correction for multiple comparisons with satisfactory *p*-values threshold in fMRI paradigms has been achieved in pigs only with deep brain stimulation (Min et al., 2012; Knight et al., 2013; Paek et al., 2015; Gibson et al., 2016; Settell et al., 2017), which is a highly invasive treatment deeply modifying brain activity. The ROI-based statistic without correction for multiple ROI comparison is a non-standard approach regarding to usual human fMRI-based statistical analyses, but was used to provide first evidence is this exploratory study. Considering the limited number of animals and the fact that they were anesthetized during the imaging procedure, detection of a specific BOLD signal in accordance with our hypotheses was already a satisfactory achievement. Of course, further studies should consider increasing the number of animals or stimulations per animal to reach a better statistical power and improving the anesthesia and/or stimulation paradigms to reduce inter-individual variability.

Even though artificial saliva is usually considered as a neutral stimulus (Hellekant et al., 1997, Clouard et al., 2014b), our results showed that it elicited a broad spectrum of brain responses in olfactogustatory brain areas, limbic and corticostriatal areas, meaning that it is not a trivial stimulation. Our approach and the global brain maps of sucrose vs. artificial saliva stimulation obtained here are comparable to those described in pigs by Clouard et al. (2014b), who investigated the impact of oral sucrose 5% stimulation on brain blood flow. Interestingly, we could also detect decreased activation in the caudate nucleus, the nucleus accumbens and the amygdala, which was reported in obese humans with sucrose stimulation compared with non-obese humans (Green et al., 2011). The global brain map of quinine vs. artificial saliva stimulation can be compared to the study of Zald et al. (2002), in which bitter stimulation promoted an increased cerebral blood flow in the dorsal cingulate cortex, the orbitofrontal cortex and the nucleus accumbens. Overall, our data are in line with a recent meta-analysis searching common pattern of activity related to basic taste stimulation, i.e., activation in the insular cortex, the thalamic region, the hippocampus, the putamen, and the cingulate cortex (Yeung et al., 2017).

Based on behavioral data available in animals (McCutcheon et al., 2012; Clouard et al., 2014a; Roura et al., 2016; Roura and Fu, 2017) and humans (Veldhuizen et al., 2006; Rudenga et al., 2010; Dalenberg et al., 2015; Field et al., 2015), the quinine vs. sucrose stimulation paradigm is now used as a common paradigm to decipher the central integration of aversive vs. pleasant stimulations using neuroimaging approaches (Zald et al., 2002; Rudenga et al., 2010; Szalay et al., 2012; van den Bosch et al., 2014; Dalenberg et al., 2015; Field et al., 2015).

The amygdala, which has direct connections from lingual nerves (King et al., 2014), is involved in pleasant and unpleasant stimuli (O’Doherty et al., 2001) and has been reported to be more activated with quinine compared to water stimulation (Zald et al., 2002). However, increased activation in amygdala with sucrose compared to quinine stimulation was also reported in humans between sucrose likers and quinine dis-likers (van den Bosch et al., 2014). In a previous study in the human, the orbitofrontal cortex, which is involved in hedonic taste valence, was found modulated by the pleasantness of the gustatory stimulation (Small et al., 2003). The anterior cingulate cortex, which is involved in taste pleasantness (Grabenhorst and Rolls, 2008), was more activated with sucrose stimulation than with quinine stimulation, which suggests a higher activation in this brain region with pleasant vs. unpleasant stimulation (Small et al., 2003; Green et al., 2015). Overall, sucrose stimulation promoted less activation in the brain than quinine stimulation, as already described in the human (Zald et al., 2002; Szalay et al., 2012). As major actors of the reward system, the nucleus accumbens and the caudate nucleus (Liu et al., 2011) were found activated with sucrose stimulation (Smith, 2004; Haase et al., 2009; Jacobson et al., 2010), but other authors showed that sucrose might promote inhibition in the nucleus accumbens in rats, whereas quinine promoted activation (Roitman et al., 2005). In our study, quinine was more effective than sucrose to induce activation in both these brain structures, but a concentration of 5% sucrose (i.e., 0.15 M) might have not been sufficient to promote a prominent response in the reward-related brain regions compared to the concentration used in the aforementioned studies (i.e., 0.64 M). Even though the limited number of animals and the use of uncorrected statistics are important limitations in our study, we managed to describe brain responses differences between quinine and sucrose stimulations in brain structures already highlighted in human studies as aforementioned. It is necessary to keep in mind that discrepancies with previous studies might be related to physiological differences in the olfaction of pigs compared to humans (Roura et al., 2016; Roura and Fu, 2017), to differences related to the tastants’ concentration (Yeung et al., 2016, 2018) and/or novelty (Clouard et al., 2012a; Kishi et al., 2017), or to the hunger/satiety status during imaging (Haase et al., 2009). Finally, it is important to remind that our animals were anesthetized, which might modulate or attenuate brain responsiveness (Hendrich et al., 2001; Willis et al., 2001).

## Conclusion

To date, among all fMRI studies performed in pigs or minipigs, this pilot study provides for the first time some preliminary evidences on brain responses to gustatory stimulation with pleasant and aversive compounds in the minipig model. Even though our study used a limited number of animals with uncorrected statistics, our overall approach can be of high interest for preclinical studies investigating the sensory integration from gustatory stimulation such as in young vs. elderly investigations (Green et al., 2011) or in pathologies such as metabolic syndrome (Green et al., 2015) or obesity (Szalay et al., 2012).

## Author Contributions

NC, MF, ER, and DV-L contributed in the experimental design. NC, EB, RJ, SQ, PM, and HS-J contributed in the technical development. NC, PM, and RJ performed the experiments. NC and PM analyzed the data. NC, HS-J, MF, ER, and DV-L wrote the manuscript.

## Conflict of Interest Statement

The authors declare that the research was conducted in the absence of any commercial or financial relationships that could be construed as a potential conflict of interest.

## References

[B1] Clouard C. Jouhanneau M. Meunier-Salaün M.-C. Malbert C. H. Val-Laillet D. (2012a). Exposures to conditioned flavours with different hedonic values induce contrasted behavioural and brain responses in pigs. *PLoS One* 7:e37968. 10.1371/journal.pone.0037968 22685528 PMC3368353

[B2] Clouard C. Loison F. Meunier-Salaün M.-C. Val-Laillet D. (2014a). An attempt to condition flavour preference induced by oral and/or postoral administration of 16% sucrose in pigs. *Physiol. Behav.* 124 107–115. 10.1016/j.physbeh.2013.10.025 24184509

[B3] Clouard C. Meunier-Salaün M.-C. Meurice P. Malbert C.-H. Val-Laillet D. (2014b). Combined compared to dissociated oral and intestinal sucrose stimuli induce different brain hedonic processes. *Front. Psychol.* 5:861. 10.3389/fpsyg.2014.00861 25147536 PMC4124794

[B4] Clouard C. Meunier-Salaün M. C. Val-Laillet D. (2012b). Food preferences and aversions in human health and nutrition: how can pigs help the biomedical research? *Animal* 6 118–136. 10.1017/S1751731111001315 22436160

[B5] Clouard C. Meunier-Salaün M.-C. Val-Laillet D. (2012c). The effects of sensory functional ingredients on food preferences, intake and weight gain in juvenile pigs. *Appl. Anim. Behav. Sci.* 138 36–46. 10.1016/j.applanim.2012.01.016

[B6] Clouard C. Val-Laillet D. (2014). Impact of sensory feed additives on feed intake, feed preferences, and growth of female piglets during the early postweaning period. *J. Anim. Sci.* 92 2133–2140. 10.2527/jas.2013-6809 24668952

[B7] Dalenberg J. R. Hoogeveen H. R. Renken R. J. Langers D. R. M. ter Horst G. J. (2015). Functional specialization of the male insula during taste perception. *Neuroimage* 119 210–220. 10.1016/j.neuroimage.2015.06.062 26142270

[B8] Danilova V. Roberts T. Hellekant G. (1999). Responses of single taste fibers and whole chorda tympani and glossopharyngeal nerve in the domestic pig, *Sus scrofa*. *Chem. Senses* 24 301–316. 10.1093/chemse/24.3.301 10400449

[B9] Duhaime A.-C. Saykin A. J. McDonald B. C. Dodge C. P. Eskey C. J. Darcey T. M. (2006). Functional magnetic resonance imaging of the primary somatosensory cortex in piglets. *J. Neurosurg. Pediatr.* 104 259–264. 10.3171/ped.2006.104.4.259 16619637

[B10] Fang M. Li J. Rudd J. A. Wai S. M. Yew J. C. C. Yew D. T. (2006). fMRI Mapping of cortical centers following visual stimulation in postnatal pigs of different ages. *Life Sci.* 78 1197–1201. 10.1016/j.lfs.2005.06.030 16182320

[B11] Fang M. Lorke D. E. Li J. Gong X. Yew J. C. C. Yew D. T. (2005). Postnatal changes in functional activities of the pig’s brain: a combined functional magnetic resonance imaging and immunohistochemical study. *Neurosignals* 14 222–233. 10.1159/000088638 16301837

[B12] Field B. A. Buck C. L. McClure S. M. Nystrom L. E. Kahneman D. Cohen J. D. (2015). Attentional modulation of brain responses to primary appetitive and aversive stimuli. *PLoS One* 10:e0130880. 10.1371/journal.pone.0130880 26158468 PMC4497686

[B13] Gibson W. S. Ross E. K. Han S. R. Van Gompel J. J. Min H.-K. Lee K. H. (2016). Anterior thalamic deep brain stimulation: functional activation patterns in a large animal model. *Brain Stimul.* 9 770–773. 10.1016/j.brs.2016.04.012 27160467 PMC5007150

[B14] Gizewski E. R. Schanze T. Bolle I. de Greiff A. Forsting M. Laube T. (2007). Visualization of the visual cortex in minipigs using fMRI. *Res. Vet. Sci.* 82 281–286. 10.1016/j.rvsc.2006.08.004 17064742

[B15] Grabenhorst F. Rolls E. T. (2008). Selective attention to affective value alters how the brain processes taste stimuli. *Eur. J. Neurosci.* 27 723–729. 10.1111/j.1460-9568.2008.06033.x 18279324

[B16] Green E. Jacobson A. Haase L. Murphy C. (2011). Reduced nucleus accumbens and caudate nucleus activation to a pleasant taste is associated with obesity in older adults. *Brain Res.* 1386 109–117. 10.1016/j.brainres.2011.02.071 21362414 PMC3086067

[B17] Green E. Jacobson A. Haase L. Murphy C. (2015). Neural correlates of taste and pleasantness evaluation in the metabolic syndrome. *Brain Res.* 1620 57–71. 10.1016/j.brainres.2015.03.034 25842372 PMC4575285

[B18] Haase L. Cerf-Ducastel B. Murphy C. (2009). Cortical activation in response to pure taste stimuli during the physiological states of hunger and satiety. *Neuroimage* 44 1008–1021. 10.1016/j.neuroimage.2008.09.044 19007893 PMC2702523

[B19] Hellekant G. Danilova V. Ninomiya Y. (1997). Primate sense of taste: behavioral and single chorda tympani and glossopharyngeal nerve fiber recordings in the rhesus monkey, *Macaca mulatta*. *J. Neurophysiol.* 77 978–993. 10.1152/jn.1997.77.2.978 9065862

[B20] Hendrich K. S. Kochanek P. M. Melick J. A. Schiding J. K. Statler K. D. Williams D. S. (2001). Cerebral perfusion during anesthesia with fentanyl, isoflurane, or pentobarbital in normal rats studied by arterial spin-labeled MRI. *Magn. Reson. Med.* 46 202–206. 10.1002/mrm.1178 11443729

[B21] Hoogeveen H. R. Dalenberg J. R. Renken R. J. ter Horst G. J. Lorist M. M. (2015). Neural processing of basic tastes in healthy young and older adults - an fMRI study. *Neuroimage* 119 1–12. 10.1016/j.neuroimage.2015.06.017 26072251

[B22] Jacobson A. Green E. Murphy C. (2010). Age-related functional changes in gustatory and reward processing regions: an fMRI study. *Neuroimage* 53 602–610. 10.1016/j.neuroimage.2010.05.012 20472070 PMC3121194

[B23] King C. T. Garcea M. Spector A. C. (2014). Restoration of quinine-stimulated Fos-immunoreactive neurons in the central nucleus of the amygdala and gustatory cortex following reinnervation or cross-reinnervation of the lingual taste nerves in rats. *J. Comp. Neurol.* 522 2498–2517. 10.1002/cne.23546 24477770 PMC4157664

[B24] Kishi M. Sadachi H. Nakamura J. Tonoike M. (2017). Functional magnetic resonance imaging investigation of brain regions associated with astringency. *Neurosci. Res.* 122 9–16. 10.1016/j.neures.2017.03.009 28366831

[B25] Knight E. J. Min H.-K. Hwang S.-C. Marsh M. P. Paek S. Kim I. (2013). Nucleus accumbens deep brain stimulation results in insula and prefrontal activation: a large animal fMRI study. *PLoS One* 8:e56640. 10.1371/journal.pone.0056640 23441210 PMC3575484

[B26] Liu X. Hairston J. Schrier M. Fan J. (2011). Common and distinct networks underlying reward valence and processing stages: a meta-analysis of functional neuroimaging studies. *Neurosci. Biobehav. Rev.* 35 1219–1236. 10.1016/j.neubiorev.2010.12.012 21185861 PMC3395003

[B27] Mäkiranta M. J. Jauhiainen J. P. T. Oikarinen J. T. Suominen K. Tervonen O. Alahuhta S. (2002). Functional magnetic resonance imaging of swine brain during change in thiopental anesthesia into EEG burst-suppression level–a preliminary study. *MAGMA* 15 27–35. 12413562 10.1007/BF02693841

[B28] McCutcheon J. E. Ebner S. R. Loriaux A. L. Roitman M. F. (2012). Encoding of aversion by dopamine and the nucleus accumbens. *Front. Neurosci.* 6:137 10.3389/fnins.2012.00137 PMC3457027 23055953

[B29] Min H.-K. Hwang S.-C. Marsh M. P. Kim I. Knight E. Striemer B. (2012). Deep brain stimulation induces BOLD activation in motor and non-motor networks: an fMRI comparison study of STN and EN/GPi DBS in large animals. *Neuroimage* 63 1408–1420. 10.1016/j.neuroimage.2012.08.006 22967832 PMC3487590

[B30] O’Doherty J. Rolls E. T. Francis S. Bowtell R. McGlone F. (2001). Representation of pleasant and aversive taste in the human brain. *J. Neurophysiol.* 85 1315–1321. 10.1152/jn.2001.85.3.1315 11248000

[B31] Paek S. B. Min H.-K. Kim I. Knight E. J. Baek J. J. Bieber A. J. (2015). Frequency-dependent functional neuromodulatory effects on the motor network by ventral lateral thalamic deep brain stimulation in swine. *Neuroimage* 105 181–188. 10.1016/j.neuroimage.2014.09.064 25451479 PMC4316813

[B32] Roitman M. F. Wheeler R. A. Carelli R. M. (2005). Nucleus accumbens neurons are innately tuned for rewarding and aversive taste stimuli, encode their predictors, and are linked to motor output. *Neuron* 45 587–597. 10.1016/j.neuron.2004.12.055 15721244

[B33] Rolls E. T. (2005). Taste, olfactory, and food texture processing in the brain, and the control of food intake. *Physiol. Behav.* 85 45–56. 10.1016/j.physbeh.2005.04.012 15924905

[B34] Rolls E. T. (2016). Functions of the anterior insula in taste, autonomic, and related functions. *Brain Cogn.* 110 4–19. 10.1016/j.bandc.2015.07.002 26277487

[B35] Roura E. Fu M. (2017). Taste, nutrient sensing and feed intake in pigs (130 years of research: then, now and future). *Anim. Feed Sci. Technol.* 233 3–12. 10.1016/j.anifeedsci.2017.08.002

[B36] Roura E. Koopmans S.-J. Lallès J.-P. Le Huerou-Luron I. de Jager N. Schuurman T. (2016). Critical review evaluating the pig as a model for human nutritional physiology. *Nutr. Res. Rev.* 29 60–90. 10.1017/S0954422416000020 27176552

[B37] Rudenga K. Green B. Nachtigal D. Small D. M. (2010). Evidence for an integrated oral sensory module in the human anterior ventral insula. *Chem. Senses* 35 693–703. 10.1093/chemse/bjq068 20595201 PMC2943409

[B38] Saikali S. Meurice P. Sauleau P. Eliat P.-A. Bellaud P. Randuineau G. (2010). A three-dimensional digital segmented and deformable brain atlas of the domestic pig. *J. Neurosci. Methods* 192 102–109. 10.1016/j.jneumeth.2010.07.041 20692291

[B39] Sauleau P. Lapouble E. Val-Laillet D. Malbert C.-H. (2009). The pig model in brain imaging and neurosurgery. *Animal* 3 1138–1151. 10.1017/S1751731109004649 22444844

[B40] Settell M. L. Testini P. Cho S. Lee J. H. Blaha C. D. Jo H. J. (2017). Functional circuitry effect of ventral tegmental area deep brain stimulation: imaging and neurochemical evidence of mesocortical and mesolimbic pathway modulation. *Front. Neurosci.* 11:104. 10.3389/fnins.2017.00104 28316564 PMC5334355

[B41] Small D. M. Gregory M. D. Mak Y. E. Gitelman D. Mesulam M. M. Parrish T. (2003). Dissociation of neural representation of intensity and affective valuation in human gustation. *Neuron* 39 701–711. 10.1016/S0896-6273(03)00467-7 12925283

[B42] Smith G. P. (2004). Accumbens dopamine mediates the rewarding effect of orosensory stimulation by sucrose. *Appetite* 43 11–13. 10.1016/j.appet.2004.02.006 15262012

[B43] Szalay C. Aradi M. Schwarcz A. Orsi G. Perlaki G. Németh L. (2012). Gustatory perception alterations in obesity: an fMRI study. *Brain Res.* 1473 131–140. 10.1016/j.brainres.2012.07.051 22885291

[B44] Val-Laillet D. (2018). Impact of food, gut-brain signals, and metabolic status on brain activity in the pig model: 10 years of nutrition research using in vivo brain imaging. *J. Anim. Sci.* (in press). 10.1017/S1751731119001745 31354119

[B45] Val-Laillet D. Aarts E. Weber B. Ferrari M. Quaresima V. Stoeckel L. E. (2015). Neuroimaging and neuromodulation approaches to study eating behavior and prevent and treat eating disorders and obesity. *Neuroimage Clin.* 8 1–31. 10.1016/j.nicl.2015.03.016 26110109 PMC4473270

[B46] van den Bosch I. Dalenberg J. R. Renken R. van Langeveld A. W. B. Smeets P. A. M. Griffioen-Roose S. (2014). To like or not to like: neural substrates of subjective flavor preferences. *Behav. Brain Res.* 269 128–137. 10.1016/j.bbr.2014.04.010 24742863

[B47] Veldhuizen M. G. van Rooden A. P. Kroeze J. H. (2006). Dissociating pleasantness and intensity with quinine sulfate/sucrose mixtures in taste. *Chem. Senses* 31 649–653. 10.1093/chemse/bjl005 16793856

[B48] Willis C. K. R. Quinn R. P. McDonell W. M. Gati J. Parent J. Nicolle D. (2001). Functional MRI as a tool to assess vision in dogs: the optimal anesthetic. *Vet. Ophthalmol.* 4 243–253. 10.1046/j.1463-5216.2001.00183.x 11906659

[B49] Yeung A. W. K. Goto T. K. Leung W. K. (2017). Basic taste processing recruits bilateral anteroventral and middle dorsal insulae: an activation likelihood estimation meta-analysis of fMRI studies. *Brain Behav.* 7:e00655. 10.1002/brb3.655 28413706 PMC5390838

[B50] Yeung A. W. K. Goto T. K. Leung W. K. (2018). Affective value, intensity and quality of liquid tastants/food discernment in the human brain: an activation likelihood estimation meta-analysis. *Neuroimage* 169 189–199. 10.1016/j.neuroimage.2017.12.034 29247808

[B51] Yeung A. W. K. Tanabe H. C. Suen J. L. K. Goto T. K. (2016). Taste intensity modulates effective connectivity from the insular cortex to the thalamus in humans. *Neuroimage* 135 214–222. 10.1016/j.neuroimage.2016.04.057 27132544

[B52] Zald D. H. Hagen M. C. Pardo J. V. (2002). Neural correlates of tasting concentrated quinine and sugar solutions. *J. Neurophysiol.* 87 1068–1075. 10.1152/jn.00358.2001 11826070

